# Complement factor C5a and C5a receptor contribute to morphine tolerance and withdrawal-induced hyperalgesia in rats

**DOI:** 10.3892/etm.2012.636

**Published:** 2012-07-10

**Authors:** YAN-HUA LI, HUA JIN, JING-SHU XU, GUANG-QIONG GUO, DA-LIN CHEN, YUN BO

**Affiliations:** 1 Departments of Anesthesiology and; 2 Oral Medicine, Kunhua Hospital, The First People’s Hospital of Yunnan Province, Kunming 650032, P.R. China

**Keywords:** morphine, tolerance, hyperalgesia, inflammation, C5a

## Abstract

Morphine is a potent opioid analgesic. However, the repeated use of morphine causes tolerance and hyperalgesia. Neuroinflammation has been reported to be involved in morphine tolerance and withdrawal-induced hyperalgesia. The complement system is a crucial effector mechanism of immune responses. The present study investigated the roles of complement factor C5a and C5a receptor (C5aR) in the development of morphine tolerance and withdrawal-induced hyperalgesia. In the present study, the levels of C5a and C5aR were increased in the L5 lumbar spinal cords of morphine-tolerant rats. The administration of C5a promoted the development of hyperalgesia and the expression of spinal antinociceptive tolerance to intrathecal morphine in both mechanical and thermal test. However, these phenomena caused by morphine were significantly attenuated by the C5aR antagonist PMX53. These results suggest that complement activation within the spinal cord is involved in morphine tolerance and withdrawal-induced hyperalgesia. C5a and C5aR may serve as novel targets for the control of morphine tolerance and withdrawal-induced hyperalgesia.

## Introduction

Morphine is a potent opioid analgesic that is widely used for clinical pain management. However, repeated administration of morphine leads to tolerance and hyperalgesia, which limit its use as an antinociceptive agent. Despite extensive research efforts in the area of morphine tolerance and hyperalgesia, the underlying mechanisms involved in these phenomena remain largely unknown. Previous studies showed that glial activation and subsequent immune responses at the lumbar spinal cord contribute to the development of morphine tolerance and withdrawal-induced hyperalgesia (1–3). Pro-inflammatory cytokines, including interleukin (IL)-1, IL-6 and tumor necrosis factor, have been implicated in the development of morphine tolerance and withdrawal-induced hyperalgesia (4,5). The complement system is a crucial effector mechanism of the innate immune system. Components of the complement cascade are constitutively produced in the central nervous system (6). However, whether the complement cascade is activated in the spinal cord under conditions of chronic morphine treatment is unknown. Furthermore, whether products of complement cascade activation modify morphine tolerance and withdrawal-induced hyperalgesia is also unknown.

The complement cascade, consisting of more than 30 soluble and membrane-bound proteins, is activated by four pathways; the classical, the alternative, the mannose-binding lectin and the extrinsic pathways (7). Eventually, all four pathways lead to the production of the important anaphylatoxin C5a in the terminal cascade (8,9). C5a exerts a range of inflammatory and immune effects by binding to the G-coupled receptor, C5aR and a second receptor, the C5a-like receptor 2 (10). The uncontrolled activation of the complement cascade causes harmful effects, including immunoparalysis and organ dysfunction (11–13). Due to its strong pro-inflammatory character, C5a is considered as the most hazardous molecule in the excessive activation of the complement cascade (13,14). A body of evidence suggests that C5a-induced C5aR activation plays a deleterious role in neural diseases including pain, Alzheimer’s disease, Huntington’s disease and amyotrophic lateral sclerosis (6,15–18). The inhibition of C5a or C5aR is beneficial for protection against these diseases in animal models. Thus, the present study was designed to evaluate the roles of C5a and C5aR in the expression of morphine tolerance and withdrawal-induced hyperalgesia in rats.

In the present study, we examined the influence of morphine administration on the expression levelss of C5 and C5aR in the L5 lumbar section of spinal cords. The effects of C5a and C5aR antagonist PMX35 on morphine tolerance and withdrawal-induced hyperalgesia were then examined.

## Materials and methods

### Animal preparation and intrathecal (i.t.) catheter implantation

Male Sprague-Dawley rats (Kunming Medical College, Kunming, China) weighing 250-300 g were used in the experiments. The rats were kept in the housing facilities for 1 week prior to experiments. The implantation of i.t. catheters was performed as described by Yaksh and Rudy (19). The rats were anesthetized with phenobarbital (50 mg/kg, intraperitoneally). The i.t. polyethylene catheters (PE-10) were inserted via an incision in the cisterna magna and advanced caudally to the lumbar enlargement of the spinal cord. The incision site was sutured in layers and the catheter was immobilized under the skin. Following surgery, all rats were returned to cages for recovery, housed individually and maintained on a 12-h light-dark cycle with food and water freely available. Rats with neurological deficit or infection were excluded from experiments. The use of experimental animals was in accordance with guidelines made by the local Committee on Animal Care and Use. The use of rats was approved by the Animal Care and Use Committees of Kunming Medical University and the First People’s Hospital of Yunnan Province.

### Behavioral tests

The antinociceptive responses were determined by mechanical (Analgesy-Meter) and thermal (tail-flick) test paradigms. The mechanical nociceptive thresholds were evaluated as described by Stein *et al* (20). Rats were gently held and increasing pressure (maximum 250 g) was applied onto the dorsal surface of the ipsilateral hind paw. The paw pressure thresholds (PPT), the pressure required to elicit paw withdrawal, were determined. The thermal nociceptive thresholds were determined by the hot water tail-flick test. The tails of rats were immersed into water (49±0.5°C) and the latency to a rapid flick was recorded. The morphine-induced antinociceptive responses in mechanical and thermal tests were expressed as the % maximum possible effect (%MPE) using the formula (21): %MPE = (WT − CT)/(CO − CT) x 100; where WT is the withdrawal latency (sec) or threshold (g) following morphine/saline treatment, CT is the latency prior to morphine/saline treatment and CO is the cut-off value (i.e. 250 g for the mechanical test and 15 sec for the tail-flick test). Behavioral tests were carried out in a blinded manner with respect to the groups.

### Experimental design

The i.t. drug administration was accomplished using a microinjection syringe connected to the i.t. catheter in conscious rats. Rats received either saline or morphine (10 mg/kg, s.c., twice daily for 5 days) and were treated once daily (11 a.m.) with C5a (10 ng, R&D Systems, Inc., Minneapolis, MN, USA), PMX53 (200 ng) or saline via i.t. catheter during induction of morphine tolerance. Chronic morphine withdrawal-induced hyperalgesia and allodynia in rats were examined 16 h after the last injection of s.c. morphine. The animals were treated with morphine (5 μg) acutely via i.t. catheter to study the expression of morphine tolerance after the recording of morphine withdrawal-induced hyperalgesia and allodynia. The acute antinociceptive activity of i.t. administered morphine in these rats was evaluated by an Analgesy-Meter and tail-flick test. The behavior recorded prior to the acute administration of i.t. morphine served as the basal latency.

### Spinal cord sample preparation

After behavioral testing, the heart perfusion of rats was performed using saline under isoflurane anesthesia. A laminectomy was performed from the lower edge of the 12th thoracic vertebra to sacral vertebra and the L5 lumbar section of the spinal cord was collected and frozen immediately in liquid nitrogen and stored at −80°C until further study.

### Enzyme-linked immunosorbent assay (ELISA)

The L5 lumbar spinal cord was collected after behavioral testing. The tissue was pooled and homogenized in homogenization buffer (phosphate-buffered saline, pH 7.4, containing 1% Triton-X100, 1 mM PMSF, 10 μg/ml aprotinin and 1 μg/ml leupeptin). Samples were spun at 15,000 x g for 30 min at 4°C. The supernatant was aliquoted and stored at −80°C for future protein quantification. The concentrations of C5a (Wuhan EIAab Science Ltd, Wuhan, China) were measured in the L5 lumbar spinal cord using specific ELISA kits according to the manufacturer’s instructions.

### Western blot analysis

L5 lumbar spinal cord samples were homogenized in ice-cold solubilizing solution [20 mM Tris-HCl (pH 7.0), 25 mM β-glycerophosphate, 2 mM EGTA, 1% Triton X-100, 1 mM vanadate, 1% aprotinin, 1 mM phenylmethylsulfonyl fluoride and 2 mM dithiothreitol] and kept on ice for 40 min. The lysate was centrifuged at 15,000 rpm for 15 min and the supernatants were collected. The protein concentrations were determined using Bio-Rad protein assay reagent (Hercules, CA, USA). Equal quantities of protein were separated by 12% SDS-polyacrylamide gel electrophoresis and transferred to a PVDF membrane (Millipore Corporation, Billerica, MA, USA). The membranes were incubated with C5aR and β-actin antibody (Santa Cruz Biotechnology, Santa Cruz, CA, USA), washed and then incubated with peroxidase-conjugated anti-mouse IgG (KPL, Gaithersburg, MD, USA). The immunoblot was revealed with an ECL western blot detection kit (Amersham Pharmacia Biotech, Buckinghamshire, UK). Densitometric analysis was performed using ImageJ software.

### Statistical analysis

All values are expressed as means ± SD. Data were analyzed by ANOVA followed by Tukey-Kramer multiple comparisons test using SPSS software (SPSS, Chicago, IL, USA). P<0.05 was considered to indicate a statistically significant result.

## Results

### Induction of C5a and C5aR at the L5 lumbar spinal cord of morphine-tolerant rats

**Figure 1:**
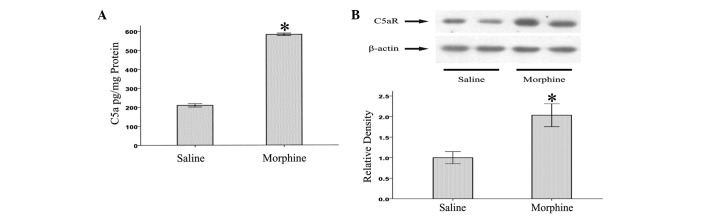
The induction of C5a and C5aR at the L5 lumbar spinal cord of morphine-tolerant rats. Rats (n=6/group) received either saline or morphine (10 mg/ kg, s.c., twice daily for 5 days). L5 lumbar spinal cords were obtained 2 h after the last morphine treatment. (A) The content of C5a at the L5 lumbar section of spinal cord was measured by ELISA. (B) The C5aR expression at the L5 lumbar section of spinal cord was measured by western blotting. Quantification is shown in the graph in the lower panel (n=6/group). Values are means ± SEM. ^*^P<0.05 vs. saline.

As an initial step in determining the association between complement factors and morphine, we examined whether the levels of C5 and C5aR were altered in the L5 lumbar spinal cords of morphine-tolerant rats. As shown in Fig. 1, we found that levels of C5a and C5aR were enhanced in the L5 lumbar spinal cords following chronic morphine treatment. This result suggests that the complement system might be involved in the pathogenesis of morphine tolerance.

### Effects of C5a and C5aR antagonist on morphine withdrawal-induced hyperalgesia and allodynia

**Figure 2:**
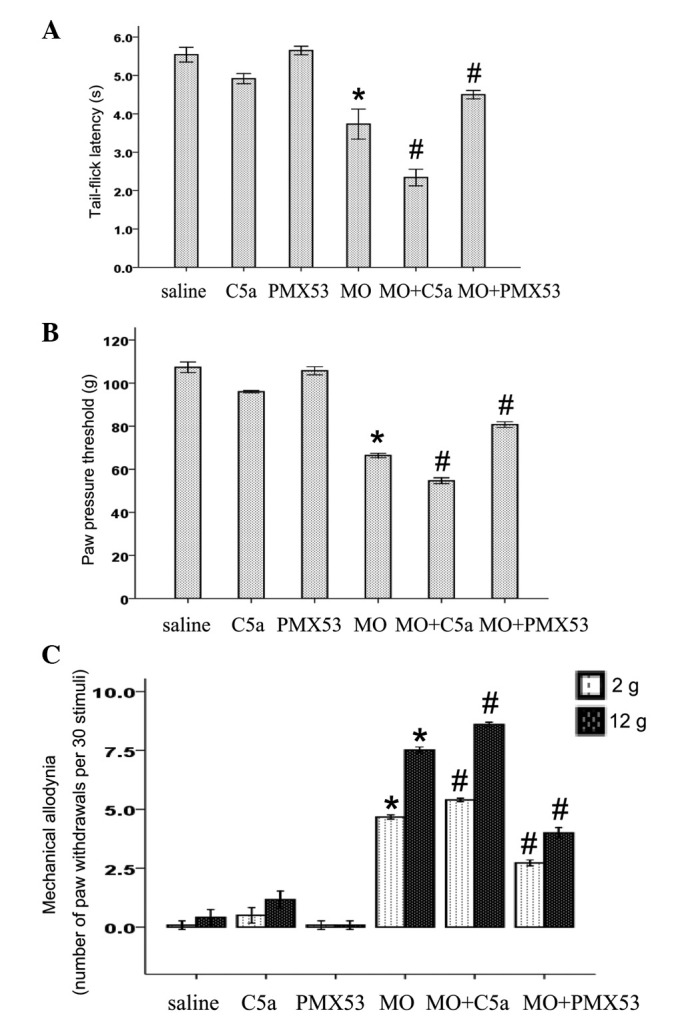
Effects of C5a and PMX53 on morphine withdrawal-induced hyperalgesia and allodynia. Rats received chronic saline, chronic C5a (10 ng), chronic PMX53 (200 ng), chronic morphine (MO), chronic morphine plus C5a (10 ng) (MO+C5a), and chronic morphine plus PMX53 (200 ng) (MO+PMX53) for 5 consecutive days. Behavioral response to noxious (A) thermal and (B and C) mechanical stimuli was recorded 16 h after the last injection of morphine or saline. Values are means ± SEM (n=12/group). *P<0.05 vs. chronic saline, ^#^P<0.05 vs. chronic morphine-treated group (MO).

In order to reveal the pathological effect of elevated levels of C5a and C5aR in morphine-tolerant rats, we investigated the effects of C5a and C5aR antagonist PMX53 on morphine withdrawal-induced hyperalgesia. As shown in Fig. 2A and B, the chronic administration of morphine (10 mg/kg, twice daily for 5 days, s.c.) led to thermal and mechanical hyperalgesia 16 h after the last injection. Similarly, the chronic administration of morphine also induced mechanical allodynia to both 2 and 12 g of mechanical stimuli (Fig. 2C). PMX53 treatment had no effect on nociceptive threshold in the saline-treated rats (Fig. 2). Chronic C5a treatment decreased nociceptive threshold in the saline-treated rats (Fig. 2). Conversely, C5a treatment during the induction of morphine tolerance significantly enhanced morphine withdrawal-induced thermal and mechanical hyperalgesia, and mechanical allodynia (Fig. 2). By contrast, the enhanced hyperalgesia induced by morphine was significantly suppresed by PMX53 (Fig. 2).

### Effects of C5a and C5aR antagonist on the expression of spinal antinociceptive tolerance to morphine

**Figure 3:**
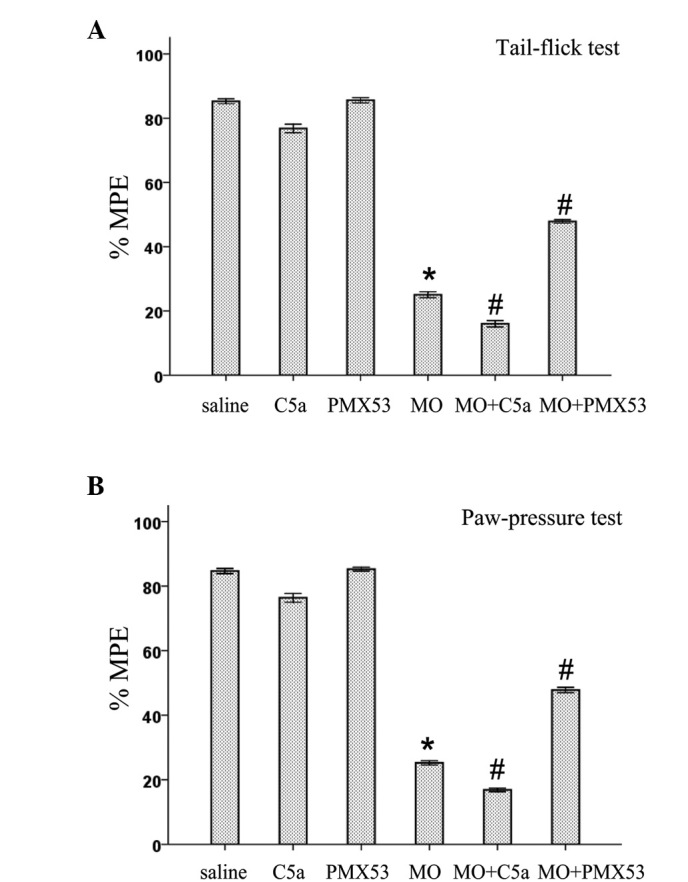
Effects of C5a and PMX53 on the expression of spinal antinociceptive tolerance to morphine. Rats received chronic saline, chronic C5a (10 ng), chronic PMX53 (200 ng), chronic morphine (MO), chronic morphine plus C5a (10 ng) (MO+C5a), and chronic morphine plus PMX53 (200 ng) (MO+PMX53) for 5 consecutive days. Acute antinociceptive acivity of i.t. morphine (5 μg) in these rats was examined using (A) a tail-flick test and (B) paw pressure test on day 6. Values are means ± SEM (n=12/group). *P<0.05 vs. chronic saline, and ^#^P<0.05 vs. chronic morphine-treated group (MO). %MPE, % maximum possible effect.

The role of C5a and C5aR in the expression of spinal antinociceptive tolerance to morphine was examined in both mechanical and thermal tests. As shown in Fig. 3, antinociceptive tolerance to acute i.t. morphine (5 μg) was induced in rats injected with saline/morphine (10 mg/kg, twice daily for 5 days, s.c.). Rats injected with PMX53 or saline had no effect on the antinociceptive activity of acute i.t. morphine (5 μg). Chronic C5a treatment slightly decreased the antinociceptive activity of acute i.t. morphine (5 μg). However, C5a treatment during the induction period of morphine tolerance (10 mg/kg, twice daily for 5 days, s.c.) significantly enhanced the expression of spinal antinociceptive tolerance to i.t. morphine (5 μg) in mechanical and thermal testing (Fig. 3). The expression of spinal antinociceptive tolerance to i.t. morphine (5 μg) was suppressed by PMX53.

## Discussion

The repeated administration of morphine is associated with significant problems, including antinociceptive tolerance and hyperalgesia (22). Accumulated evidence suggests mediators underlying morphine tolerance and hyperalgesia. However, effective pharmacotherapy has not been established for morphine-induced side effects. Previous studies proposed the association between immune responses in the spinal cord and morphine tolerance and withdrawal-induced hyperalgesia (21,23,24). The complement system may be a crucial regulator of morphine tolerance and withdrawal-induced hyperalgesia, as activation of complement cascade results in the enhancement of immune responses. In the present study, the main finding is the identification of C5a and C5aR as a likely promoting mediator for morphine tolerance and withdrawal-induced hyperalgesia.

The complement system is known to be a potent effector mechanism of the immune system. In the spinal cord, neuron, microglia and astrocytes constitutively produce complement components, including C5a and C5aR (25,26). Hence, there is potential for rapid activation of the complement cascade. Microglia and astrocytes may enhance the level of complement components under various situations, including central nervous system infection, brain injury and pain (6,17,27,28). The present results demonstrate that the levels of C5a and C5aR were increased in the L5 lumbar spinal cords of morphine-tolerant rats. However, it is still unknown in which type(s) of these cells that upregulated levels of C5a and C5aR occur. These results suggest that the activation of the complement cascade is involved in the development of morphine tolerance and withdrawal-induced hyperalgesia.

The increased levels of C5a and C5aR in morphine-tolerant rats suggest that the pathological role of the upregulation of C5a and C5aR in morphine tolerance and withdrawal-induced hyperalgesia requires elucidation. Although the complement system plays a crucial role in host defense in times of immunological challenge, the over-activation of the complement cascade causes deleterious effects on the host as well. C5a/C5aR inhibitors have shown efficacy in controlling the pathogenesis of inflammation-associated diseases, including autoimmune diseases and pain (25,29–31). The present results demonstrate that C5a treatment promoted the development of morphine tolerance and withdrawal-induced hyperalgesia in rats. However, the C5aR antagonist PMX53 blocked the development of these phenomena. Thus, C5a and C5aR may play promoting roles in the development of morphine tolerance and hyperalgesia.

Spinal inflammatory immune responses contribute to the mechanisms of morphine tolerance. Several studies have demonstrated that pro-inflammatory cytokines IL-1β, IL-6 and tumor necrosis factor (TNF)-α enhances the expression of morphine tolerance and withdrawal-induced hyperalgesia in rats (1,4,23). The pivotal role of the activation of NF-κB in inflammation during the induction of morphine tolerance has also been well elucidated (32,33). C5a activates and attracts astrocytes and microglia, and increases levels of pro-inflammatory cytokines (34–36). It has also been reported that C5a binds to neurons to enhance the level of intracellular calcium and activate NF-κB (37). Therefore, activation of C5a and C5aR may be part of a pathway to promote the development of morphine tolerance and withdrawal-induced hyperalgesia.

In summary, this is the first demonstration that the complement cascade is activated in the spinal cord by repeated injection of morphine, and that C5a and C5aR regulate morphine tolerance and withdrawal-induced hyperalgesia. A more detailed analysis in future studies may be useful to design an effective therapeutic strategy for morphine tolerance and withdrawal-induced hyperalgesia.

## References

[1] Raghavendra V, Rutkowski MD, DeLeo JA (2002). The role of spinal neuroimmune activation in morphine tolerance/hyperalgesia in neuropathic and sham-operated rats. J Neurosci.

[2] Horvath RJ, Romero-Sandoval EA, De Leo JA (2010). Inhibition of microglial P2X4 receptors attenuates morphine tolerance, Iba1, GFAP and mu opioid receptor protein expression while enhancing perivascular microglial ED2. Pain.

[3] Ramos KM, Lewis MT, Morgan KN (2010). Spinal upregulation of glutamate transporter GLT-1 by ceftriaxone: therapeutic efficacy in a range of experimental nervous system disorders. Neuroscience.

[4] Johnston IN, Milligan ED, Wieseler-Frank J (2004). A role for proinflammatory cytokines and fractalkine in analgesia, tolerance, and subsequent pain facilitation induced by chronic intrathecal morphine. J Neurosci.

[5] Hutchinson MR, Bland ST, Johnson KW, Rice KC, Maier SF, Watkins LR (2007). Opioid-induced glial activation: mechanisms of activation and implications for opioid analgesia, dependence, and reward. ScientificWorldJournal.

[6] Woodruff TM, Ager RR, Tenner AJ, Noakes PG, Taylor SM (2010). The role of the complement system and the activation fragment C5a in the central nervous system. Neuromolecular Med.

[7] Kemper C, Atkinson JP (2007). T-cell regulation: with complements from innate immunity. Nat Rev Immunol.

[8] Ehrnthaller C, Ignatius A, Gebhard F, Huber-Lang M (2011). New insights of an old defense system: structure, function, and clinical relevance of the complement system. Mol Med.

[9] Ricklin D, Hajishengallis G, Yang K, Lambris JD (2010). Complement: a key system for immune surveillance and homeostasis. Nat Immunol.

[10] Huber-Lang MS, Riedeman NC, Sarma JV (2002). Protection of innate immunity by C5aR antagonist in septic mice. FASEB J.

[11] Recknagel S, Bindl R, Kurz J (2011). C5aR-antagonist significantly reduces the deleterious effect of a blunt chest trauma on fracture healing. J Orthop Res.

[12] Hecke F, Schmidt U, Kola A, Bautsch W, Klos A, Kohl J (1997). Circulating complement proteins in multiple trauma patients – correlation with injury severity, development of sepsis, and outcome. Crit Care Med.

[13] Ganter MT, Brohi K, Cohen MJ (2007). Role of the alternative pathway in the early complement activation following major trauma. Shock.

[14] Gerard C (2003). Complement C5a in the sepsis syndrome – too much of a good thing?. N Engl J Med.

[15] Liang DY, Li X, Shi X (2011). The complement component C5a receptor mediates pain and inflammation in a postsurgical pain model. Pain.

[16] Jacob A, Hack B, Bai T, Brorson JR, Quigg RJ, Alexander JJ (2010). Inhibition of C5a receptor alleviates experimental CNS lupus. J Neuroimmunol.

[17] Jang JH, Liang D, Kido K, Sun Y, Clark DJ, Brennan TJ (2011). Increased local concentration of complement C5a contributes to incisional pain in mice. J Neuroinflammation.

[18] Fonseca MI, Ager RR, Chu SH (2009). Treatment with a C5aR antagonist decreases pathology and enhances behavioral perfor mance in murine models of Alzheimer’s disease. J Immunol.

[19] Yaksh TL, Rudy TA (1976). Analgesia mediated by a direct spinal action of narcotics. Science.

[20] Stein C, Gramsch C, Herz A (1990). Intrinsic mechanisms of antinociception in inflammation: local opioid receptors and beta-endorphin. J Neurosci.

[21] Raghavendra V, Tanga FY, DeLeo JA (2004). Attenuation of morphine tolerance, withdrawal-induced hyperalgesia, and associated spinal inflammatory immune responses by propentofylline in rats. Neuropsychopharmacology.

[22] Kamei J, Ohsawa M, Hayashi SS, Nakanishi Y (2011). Effect of chronic pain on morphine-induced respiratory depression in mice. Neuroscience.

[23] Song P, Zhao ZQ (2001). The involvement of glial cells in the development of morphine tolerance. Neurosci Res.

[24] Hutchinson MR, Lewis SS, Coats BD (2010). Possible involvement of toll-like receptor 4/myeloid differentiation factor-2 activity of opioid inactive isomers causes spinal proinflammation and related behavioral consequences. Neuroscience.

[25] Rus H, Niculescu F (2001). The complement system in central nervous system diseases. Immunol Res.

[26] Rus H, Cudrici C, David S, Niculescu F (2006). The complement system in central nervous system diseases. Autoimmunity.

[27] Liu L, Aldskogius H, Svensson M (1998). Ultrastructural localization of immunoglobulin G and complement C9 in the brain stem and spinal cord following peripheral nerve injury: an immunoelectron microscopic study. J Neurocytol.

[28] Davoust N, Jones J, Stahel PF, Ames RS, Barnum SR (1999). Receptor for the C3a anaphylatoxin is expressed by neurons and glial cells. Glia.

[29] Twining CM, Sloane EM, Schoeniger DK (2005). Activation of the spinal cord complement cascade might contribute to mechanical allodynia induced by three animal models of spinal sensitization. J Pain.

[30] Rioux P (2001). TP-10 (AVANT Immunotherapeutics). Curr Opin Investig Drugs.

[31] Shen Y, Meri S (2003). Yin and Yang: complement activation and regulation in Alzheimer’s disease. Prog Neurobiol.

[32] Sun T, Song WG, Fu ZJ, Liu ZH, Liu YM, Yao SL (2006). Alleviation of neuropathic pain by intrathecal injection of antisense oligo-nucleotides to p65 subunit of NF-kappaB. Br J Anaesth.

[33] Wang Z, Ma W, Chabot JG, Quirion R (2010). Calcitonin gene-related peptide as a regulator of neuronal CaMKII-CREB, microglial p38–NFkappaB and astroglial ERK-Stat1/3 cascades mediating the development of tolerance to morphine-induced analgesia. Pain.

[34] Ischenko A, Sayah S, Patte C (1998). Expression of a functional anaphylatoxin C3a receptor by astrocytes. J Neurochem.

[35] O’Barr S, Cooper NR (2000). The C5a complement activation peptide increases IL-1beta and IL-6 release from amyloid-beta primed human monocytes: implications for Alzheimer’s disease. J Neuroimmunol.

[36] Osaka H, McGinty A, Hoepken UE, Lu B, Gerard C, Pasinetti GM (1999). Expression of C5a receptor in mouse brain: role in signal transduction and neurodegeneration. Neuroscience.

[37] O’Barr SA, Caguioa J, Gruol D (2001). Neuronal expression of a functional receptor for the C5a complement activation fragment. J Immunol.

